# Supplementary material for: The Promise and Challenges of Cyclic Dinucleotides as Molecular Adjuvants for Vaccine Development
Source: Vaccines (Basel). 2021 Aug 17;9(8):917. doi: 10.3390/vaccines9080917 (PMC8402453; doi:10.3390/vaccines9080917)
Supplement: Supplementary file 1 [file vaccines-09-00917-s001.zip › vaccines-1297039-supplementary.pdf]

The promise and challenges of cyclic dinucleotides as molecular adjuvants for vaccine development

Hongbin Yan [1,*] and Wangxue Chen [2,3,*]

1 Department of Chemistry, Brock University, St. Catharines, ON L2S 3A1, Canada
2 Human Health and Therapeutics Research Centre, National Research Council Canada, Ottawa, ON K1A 0R6, Canada
3 Department of Biological Sciences, Brock University, St. Catharines, ON L2S 3A1, Canada
* Correspondence: tyan@brocku.ca (H.Y.); Wangxue.Chen@nrc.gc.ca (W.C.)

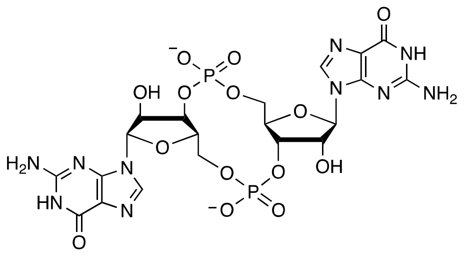

c-di-GMP (1-11)

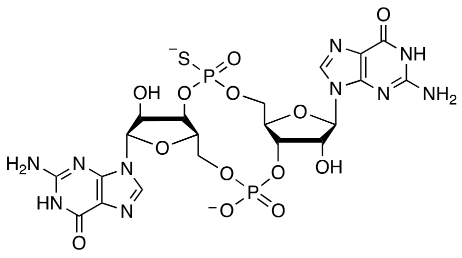

c-di-GMP-S1 (1, 12, 13)

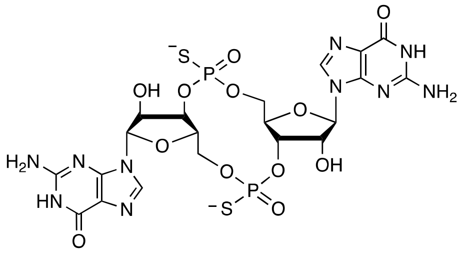

c-di-GMP-S2 (7, 13, 14)

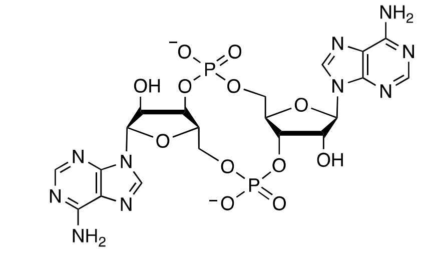

c-di-AMP (1, 4, 8, 9, 15)

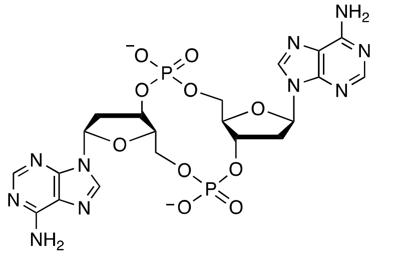

c-di-dAMP (16)

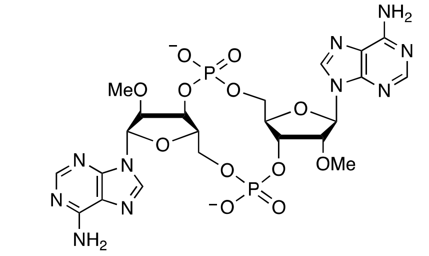

2′-MeO-c-di-AMP (16)

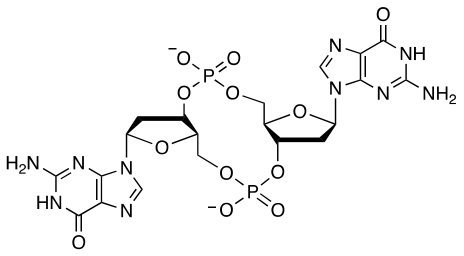

c-di-dGMP (1)

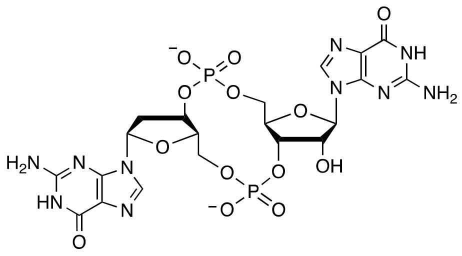

cdGpGp (1, 17)

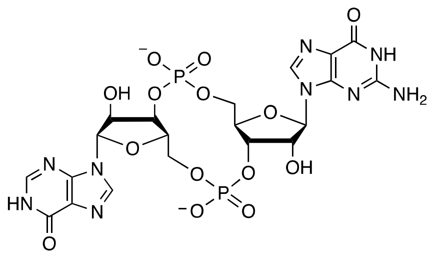

cIpGp (1)

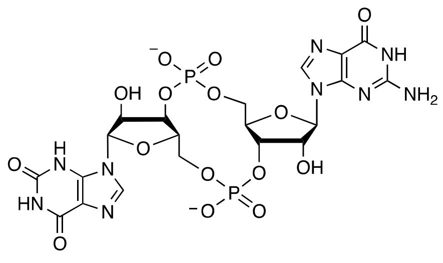

cXpGp (1)

Figure S1. Selected CDNs containing 3′-5′ internucleotide phosphate/phosphorothioate linkages reported in the literature.

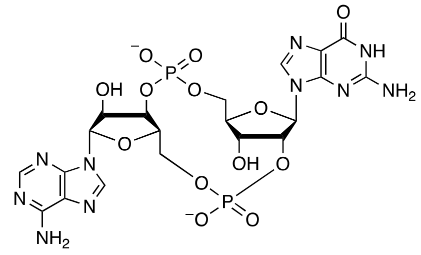

2′3′-cGAMP (24)

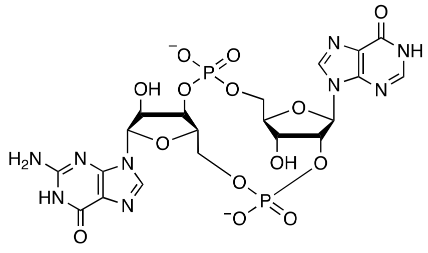

2′3′-cIGMP (20)

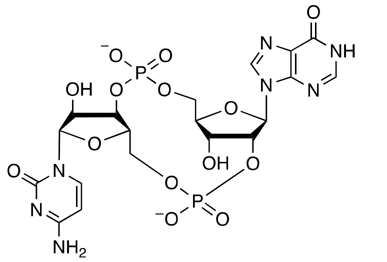

2′3′-cICMP (20)

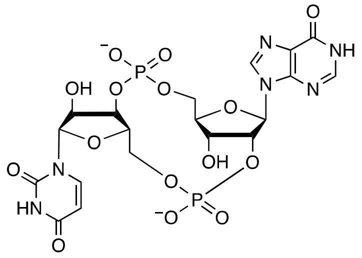

2′3′-cIUMP (20)

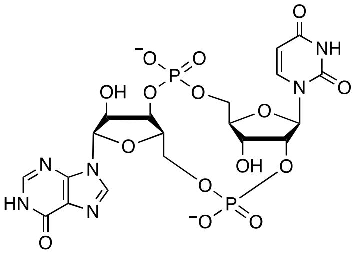

2′3′-cUIMP (20)

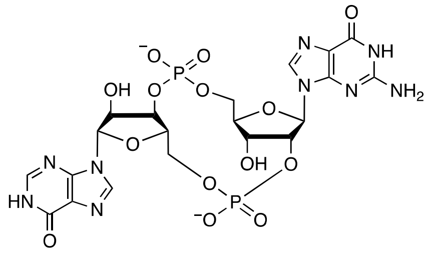

2′3′-cGIMP (20)

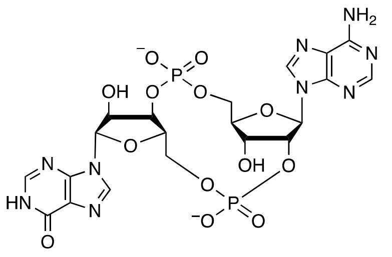

2′3′-cAIMP (19, 20)

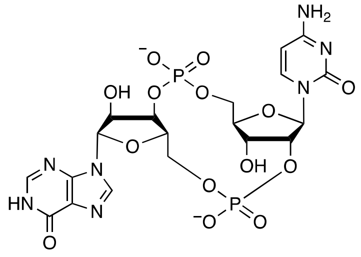

2′3′-cCIMP (20)

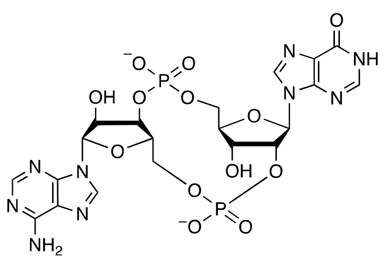

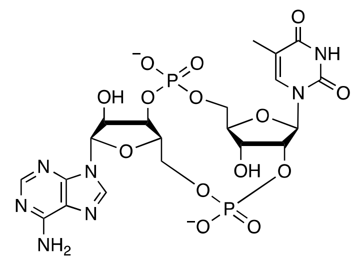

Figure S2. Selected CDNs containing 2′-5′ and both 2′-5′ and 3′-5′ internucleotide phosphate/phosphorothioate linkages reported in the literature.

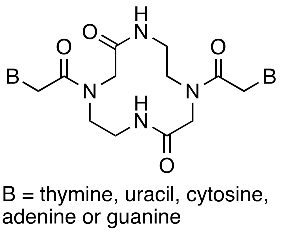

PNA backbone (27)

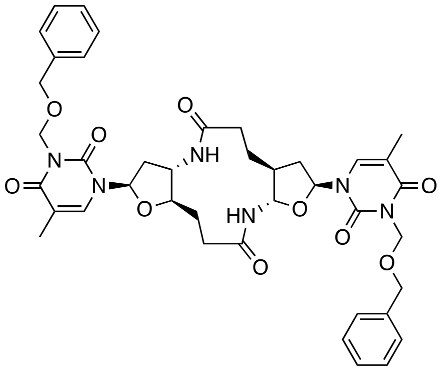

Amide backbone (28)

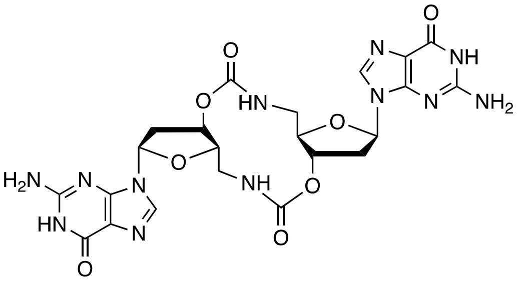

Carbamate backbone (29)

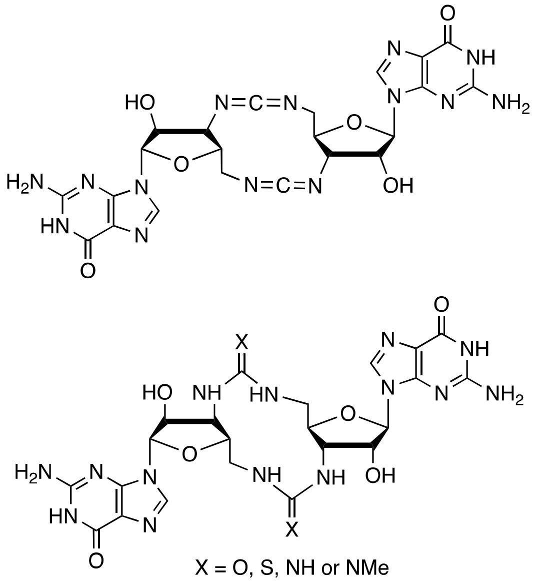

Carbodiimide, urea, thiourea and guanidinium backbone (30)

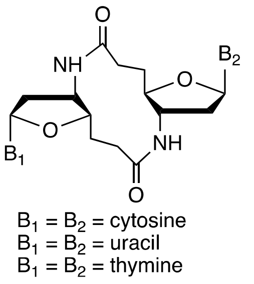

Amide backbone (31)

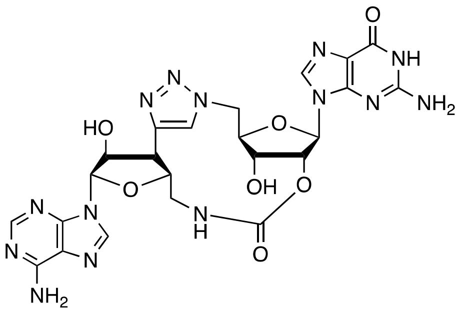

Triazolyl backbone (32)

Figure S3. Selected CDN analogs with internucleotide linkages that are not phosphorous based.

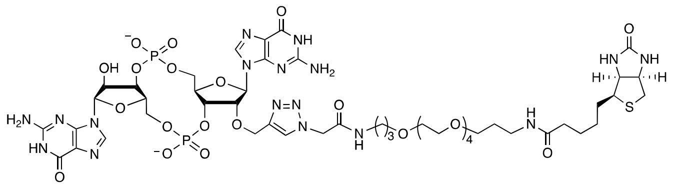

Biotin-c-di-GMP (33)

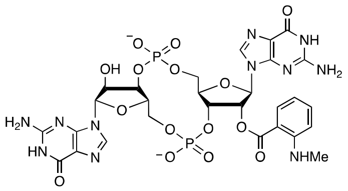

Mant-(c-di-GMP) (34)

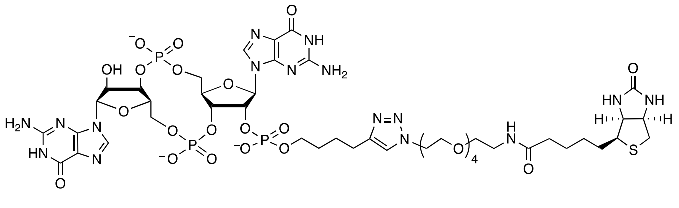

Biotin-c-di-GMP (8)

Figure S4. c-di-GMP conjugates reported.

References

1. Ross P, Mayer R, Weinhouse H, Amikam D, Huggirat, Y., Benziman, M.; et al. The cyclic diguanylic acid regulatory system of cellulose synthesis in acetobacter-xylinum - chemical synthesis and biological-activity of cyclic-nucleotide dimer, trimer, and phosphothioate derivatives. Journal of Biological Chemistry. 1990;265(31):18933-43.
2. Hayakawa Y, Nagata R, Hirata A, Hyodo, M., Kawai, R. A facile synthesis of cyclic bis(3′-> 5′)diguanylic acid. Tetrahedron. 2003;59(34):6465-71.
3. Yan HB, Aguilar AL. Synthesis of 3′,5′-cyclic diguanylic acid (cdiGMP) using 1-(4-chlorophenyl)-4-ethoxypiperidin-4-yl as a protecting group for 2′-hydroxy functions of ribonucleosides. Nucleosides Nucleotides & Nucleic Acids. 2007;26(2):189-204.
4. Amiot, N., Heintz, K., Giese, B. New approach for the synthesis of c-di-GMP and its analogues. Synthesis-Stuttgart. 2006(24):4230-6.
5. Kiburu I, Shurer A, Yan L, Sintim HO. A simple solid-phase synthesis of the ubiquitous bacterial signaling molecule, c-di-GMP and analogues. Molecular Biosystems. 2008;4(6):518-20.
6. Ching SM, Tan WJ, Chua KL, Lam YL. Synthesis of cyclic di-nucleotidic acids as potential inhibitors targeting diguanylate cyclase. Bioorganic & Medicinal Chemistry. 2010;18(18):6657-65.
7. Gaffney BL, Veliath, E., Zhao JW, Jones RA. One-flask syntheses of c-di-GMP and the [*R*p,*R*p] and [*R*p,*S*p] thiophosphate analogues. Organic Letters. 2010;12(14):3269-71.
8. Gaffney BL, Stebbins ND, Jones RA. Synthesis of biotinylated c-di-GMP And c-di-AMP using Click conjugation. Nucleosides Nucleotides & Nucleic Acids. 2013;32(1):1-16.
9. Wang CH, Sinn M, Stifel J, Heiler AC, Sommershof, A., Hartig JS. Synthesis of all possible canonical (3′-5′-linked) cyclic dinucleotides and evaluation of riboswitch interactions and immune-stimulatory effects. Journal of the American Chemical Society. 2017;139(45): 16154-60.
10. Tsukamoto, M., Oyama, K. Recent application of acidic 1,3-azolium salts as promoters in the solution-phase synthesis of nucleosides and nucleotides. Tetrahedron Letters. 2018;59(26):2477-84.
11. Grajkowski A, Takahashi M, Kaczynski T, Srivastava SC, Beaucage SL. An improved phosphoramidite approach for the chemical synthesis of 3′,5′-cyclic diguanylic acid. Tetrahedron Letters. 2019;60(5):452-5.
12. Hyodo, M., Sato, Y., Hayakawa, Y. Synthesis of cyclic bis(3′-5′)diguanylic acid (c-di-GMP) analogs. Tetrahedron. 2006;62(13):3089-94.
13. Yan HB, Wang XL, KuoLee, R., Chen WX. Synthesis and immunostimulatory properties of the phosphorothioate analogues of cdiGMP. Bioorganic & Medicinal Chemistry Letters. 2008;18(20):5631-4.
14. Knouse KW, deGruyter JN, Schmidt MA, Zheng B, Vantourout JC, Kingston, C.; et al. Unlocking P(V): Reagents for chiral phosphorothioate synthesis. Science. 2018;361(6408): 1234-8.

15. Suzuki N, Oyama K, Tsukamoto, M. Practical synthesis of cyclic bis(3′-5′)diadenylic acid (c-di-AMP). Chemistry Letters. 2011;40(10):1113-4.
16. Tezuka T, Suzuki N, Ishida K, Oyama K, Aoki S, Tsukamoto, M. Synthesis of 2′-modified cyclic bis(3′-5′)diadenylic acids (c-di-AMPs) and their promotion of cell division in a freshwater green alga. Chemistry Letters. 2012;41(12):1723-5.
17. Mano E, Hyodo M, Sato Y, Ishihara, Y., Ohta, M., Hayakawa, Y. Synthesis of cyclic bis(3′-5′)-2′-deoxyguanylic/guanylic acid (c-dGpGp) and its biological activities to microbes. Chemmedchem. 2007;2(10):1410-3.
18. Li J, Lee RK, Chen WX, Yan HB. 2′-Fluoro-c-di-GMP as an oral vaccine adjuvant. RSC Advances. 2019;9(71):41481-9.
19. Lioux T, Mauny MA, Lamoureux A, Bascoul N, Hays M, Vernejoul, F.; et al. Design, synthesis, and biological evaluation of novel cyclic adenosine-inosine monophosphate (cAIMP) analogs that activate stimulator of interferon genes (STING). Journal of Medicinal Chemistry. 2016;59(22):10253-67.
20. Wang ZH, Zhao CC, Wang CL, Zhang H, Ma DJ, Zhang QZ; et al. Synthesis and biological evaluation of all possible inosine-mixed cyclic dinucleotides that activate different hSTING variants. Bioorganic & Medicinal Chemistry. 2021;29: 115899.
21. Roembke BT, Zhou J, Zheng Y, Sayre D, Lizardo, A., Bernard, L.; et al. A cyclic dinucleotide containing 2-aminopurine is a general fluorescent sensor for c-di-GMP and 3′,3′-cGAMP. Molecular Biosystems. 2014;10(6):1568-75.
22. Shiraishi K, Saito-Tarashima N, Igata Y, Murakami K, Okamoto, Y., Miyake, Y.; et al. Synthesis and evaluation of c-di-4′-thioAMP as an artificial ligand for c-di-AMP riboswitch. Bioorganic & Medicinal Chemistry. 2017;25(14):3883-9.
23. Shchokolova AS, Rymko AN, Kvach SV, Shabunya PS, Fatykhava SA, Zinchenko AI. Enzymatic synthesis of 2′-ara and 2′-deoxy analogues of c-di-GMP. Nucleosides Nucleotides & Nucleic Acids. 2015;34(6):416-23.
24. Zhang X, Shi HP, Wu JX, Zhang XW, Sun LJ, Chen, C.; et al. Cyclic GMP-AMP containing mixed phosphodiester linkages is an endogenous high-affinity ligand for STING. Molecular Cell. 2013;51(2):226-35.
25. Xie, X., Liu JY, Wang XW. Design, Synthesis and biological evaluation of (2′,5′ and 3′5′-linked) cGAMP analogs that activate stimulator of interferon genes (STING). Molecules. 2020;25(22): 5285.
26. Corrales L, Glickman LH, McWhirter SM, Kanne DB, Sivick KE, Katibah GE; et al. Direct activation of STING in the tumor microenvironment leads to potent and systemic tumor regression and immunity. Cell Reports. 2015;11(7):1018-30.
27. Verheijen JC, Grotenbreg GM, de Ruyter LH, van der Klein PAM, van der Marel GA, van Boom JH. An expeditious liquid-phase synthesis of cyclic peptide nucleic acids. Tetrahedron Letters. 2000;41(20):3991-5.
28. Chakraborty TK, Koley D, Prabhakar S, Ravi R, Kunwar AC. Synthesis and conformational studies of amide-linked cyclic homooligomers of a thymidine-based nucleoside amino acid. Tetrahedron. 2005;61(40):9506-12.
29. Kline T, Jackson SR, Deng W, Verlinde, C., Miller SI. Design and synthesis of bis-carbamate analogs of cyclic bis-(3′-5′)-diguanylic acid (c-di-GMP) and the acyclic dimer PGPG. Nucleosides Nucleotides & Nucleic Acids. 2008;27(12):1282-300.
30. Gaffney BL, Jones RA. Synthesis of c-di-GMP analogs with thiourea, urea, carbodiimide, and guanidinium linkages. Organic Letters. 2014;16(1):158-61.
31. Pal, C., Chakraborty TK. Synthesis of amide-linked cyclic dinucleotide analogues with pyrimidine bases. Asian Journal of Organic Chemistry. 2017;6(10):1421-7.
32. Dialer CR, Stazzoni S, Drexler DJ, Muller FM, Veth, S., Pichler, A.; et al. A Click-chemistry linked 2′3′-cGAMP analogue. Chemistry – A European Journal. 2019;25(8):2089-95.
33. Grajkowski A, Cieslak J, Gapeev A, Schindler, C., Beaucage SL. Convenient synthesis of a propargylated cyclic (3′-5′) diguanylic acid and its "Click" conjugation to a biotinylated azide. Bioconjugate Chemistry. 2010;21(11):2147-52.
34. Sharma IM, Dhanaraman T, Mathew R, Chatterji, D. Synthesis and characterization of a fluorescent analogue of cyclic di-GMP. Biochemistry. 2012;51(27):5443-53.
